# Multimorbidity patterns: obesity as the main modifiable risk factor in adult women in Southern Brazil

**DOI:** 10.20945/2359-3997000000642

**Published:** 2023-05-29

**Authors:** Débora Luiza Franken, Juvenal Soares Dias-da-Costa, Maria Teresa Anselmo Olinto, Jaqueline Sturmer, Rafaela Balzaretti Bordin, Vera Maria Vieira Paniz

**Affiliations:** 1 Universidade do Vale do Rio dos Sinos São Leopoldo RS Brasil Programa de Pós-graduação em Saúde Coletiva, Universidade do Vale do Rio dos Sinos, São Leopoldo, RS, Brasil; 2 Universidade Federal do Rio Grande do Sul Faculdade de Medicina Porto Alegre RS Brasil Faculdade de Medicina, Universidade Federal do Rio Grande do Sul, Porto Alegre, RS, Brasil

**Keywords:** Multimorbidity, multiple chronic conditions, obesity, risk factor, health surveys

## Abstract

**Objective:**

To identify multimorbidity patterns in women in southern Brazil, and its relationship with sociodemographic, lifestyle characteristics, and nutritional status, as well as to explore the main independent risk factor for the identified patterns.

**Subjects and methods:**

This is a cross-sectional, population-based study with 1,128 women (20-69 years), southern Brazil. Chronic conditions were identified using the therapeutic and chemical anatomical classification of continuous use of medications. Multimorbidity was assessed as ≥2 or ≥3 chronic conditions to identify dyads and triads. Poisson regression was used to explore risk factors in the different adjustment models. As independent variables evaluated, in addition to sociodemographic characteristics, lifestyle variables were included: consumption of fruits and vegetables, physical activity, alcohol consumption, smoking and nutritional status.

**Results:**

Eleven dyads (frequencies between 2.0% and 6.4%) and three triads (frequencies between 1.9% and 2.1%) of morbidities were identified in the study. Aging was related to a higher prevalence of all patterns, and obesity was a risk factor for multimorbidity patterns that contained conditions related to the cardiovascular and metabolic system and mental health. After adjustment, obesity increased the probability of “hypertension + common mental disorders (CMD)” (PR 3.63; 95% CI 1.94-6.78) and “dyslipidemia + CMD” (PR 3.69; 95% CI 1.08-12.65) by more than three times.

**Conclusion:**

This study identified common and important diseases in the patterns, associated with a common risk factor, obesity, that must be addressed by public health policies to prevent multimorbidity.

## INTRODUCTION

Chronic health problems represent the dominant burden of health care today, especially because they are strongly related to population aging (1). Globally, chronic non-communicable diseases (CNCDs) have high rates of mortality and morbidity (2,3), besides to being considered the greatest source of disease burden and responsible for the highest health costs (1). Furthermore, evidence has shown that, on a daily basis, the reality of people is even more complicated, since it is common for them to be affected by more than one chronic health condition, presenting itself as a public health problem of complex management, both in developed and developing countries (4).

Multimorbidity is characterized by coexistence of different chronic conditions in an individual without a single condition being considered as the main one, enhancing the idea of comprehensive care (5,6). Its operational definition differs in literature, with the occurrence of two or more chronic diseases being the most commonly used cutoff point, mainly in the adult population, in which the combination of more than two conditions is less prevalent (5–7). Other approaches include counting the number of chronic conditions, clusters of pre-established diseases, and identification of multimorbidity patterns from the list of the most prevalent diseases (8–10). Regarding its prevalence, studies have shown that multimorbidity affects the majority of the elderly population and women (11–13). A longitudinal study with a representative sample of Brazilian adults revealed that the prevalence of multimorbidity was 67.8% when considered ≥ 2 chronic conditions and 47.1% when considered ≥ 3 (13).

The available literature is inconsistent with regard to identifying different combinations of chronic conditions and their magnitude. Certain multimorbidity patterns can have a different impact on the health of affected individuals, both in terms of quality of life and mortality, in addition to influencing the use of health services differently and, consequently, the resulting costs (9,14–16). In Brazil, direct and indirect costs related to the group of cardiovascular diseases totaled R$ 37.1 billion in 2015, representing an estimated average cost of 0.7% of the Brazilian GDP (17). Furthermore, there are still gaps in the literature regarding the risk factors for multimorbidity patterns. Existing studies reveal that, in addition to the wide variety of multimorbidity patterns that can be found, these are mainly associated with different sociodemographic characteristics of the population and also according to the lifestyle characteristics of each individual (15,18). In addition, obesity has an established role as a risk factor for several CNCDs, and studies have already shown its dose-response association with an increase in the number of chronic conditions (19,20). In this sense, the identification of modifiable risk factors involved in the multi-causality of multimorbidity patterns is essential for understanding the magnitude and transcendence of this problem in the population of adult women, in order to propose effective therapeutic and prevention strategies for this population.

Thus, this study aimed to identify multimorbidity patterns in women in southern Brazil, and its relationship with sociodemographic, lifestyle, and nutritional status characteristics, as well as to explore the main independent risk factor for the identified patterns.

## SUBJECTS AND METHODS

### Study design and population

This cross-sectional study included women aged 20-69 years living in the urban area of São Leopoldo, a municipality in southern Brazil. Data were collected in 2015 (from February to October), and all women in the age group of interest, except for pregnant women residing in the selected households, were eligible for the study. The larger project of which this study is a part, was submitted to and approved by the Ethics and Research Committee of the University of Vale do Rio dos Sinos (CAAE 30872914.6.0000.5344, Protocol 650.443), and all participants provided written informed consent.

The sample size calculation for the larger study was based on the prevalence of various outcomes related to women's health. A power of 80% was considered to identify a risk ratio of 2.0 between women with lower and higher education (not exposed – with 15 years or more of education) with a confidence level of 95%. Finally, 10% was added for eventual losses/refusals, and 15% was added to control for confounding factors in the data analysis, resulting in a total sample size of 1,281 women.

Sampling was probabilistic and performed at multiple stages. First, 371 census sectors in the urban area of São Leopoldo were listed and classified based on the highest average monthly nominal income for persons aged 10 years or older (with or without income). The sectors were numbered from 1 to 371, according to this classification. Subsequently, 45 census sectors were randomly selected. So, considering the average number of women per household (0.93), as well as the proportion of women in the age group of interest (32.1%), we estimate that 1,613 households would be visited. This number was divided into 45 census sectors, totaling 36 households in each sector (21). Posteriorly, was to draw randomly, in each conglomerate, the block and the corner to start the research. Households were selected from the defined point (corner indicated for the beginning), always toward the left of those facing the initial corner; the first house was selected for the study, skipping two houses, selecting the fourth house again, and so on, until 36 households per sector were completed (22).

Trained interviewers collected data using a standardized and pretested questionnaire in a pilot study, which took place in a census sector not included among those selected for the study. Data quality control was carried out through a reduced questionnaire administered to 10% of the participants.

### Dependent variable

Twenty-six chronic conditions were identified in the study population, with the most prevalents being: arterial hypertension, common mental disorders (CMDs), acid-related digestive disorders, dyslipidemia, thyroid diseases, diabetes mellitus (DM), circulatory disorders, and chronic pain (23). Information about their presence was obtained from the identification of the continuous use of medicines prescribed by a doctor, reported by the women at the time of the interview. The Anatomical Therapeutic Chemical (ATC) classification (24) was used to classify drugs according to the organ or system in which they operated, thus serving as a proxy for the identification of chronic conditions (23,25). When the same drug had more than one indication of use, the participant's information on the reason why the medicine was prescribed was considered. We excluded drugs reported as being occasional use from the outcome assessment. Multimorbidity was categorized for participants with two or three or more chronic conditions (5–7), and the different combinations of these defined the patterns as dyads and triads of chronic conditions.

### Exposures

The evaluated exposures were:

- Sociodemographic: age collected in years (categorized every 10 years), self-reported skin color (white, black, indigenous, yellow and brown), marital status (not having a partner and having a partner), education in years (≥11 years; eight to 10 years; five to seven years; ≤4 years), household income per capita in quartiles (the first quartile, ≤R$ 525.30; the second quartile, >R$ 525.30-R$ 869.00; the third quartile, >R$ 869.00-R$ 1547.00; and the fourth quartile, >R$ 1547.00) economic class (A/B; C; D/E) according to the economic classification criteria proposed by the Brazilian Association of Research Companies (26), and occupation (employed and unemployed);
- Lifestyle: smoking (non-smoker, former smoker, current smoker), alcohol consumption based on frequency, type of drink, and quantity ingested, considering the risk of alcohol consumption for women and the cutoff point of 15 g of ethanol/d (27) (no/yes), physical activity (active and inactive), and consumption of fruits and vegetables (adequate and inadequate). Participants were considered physically active when they reached at least 150 minutes of weekly physical activity, as verified by the International Physical Activity Questionnaire short version (28). The consumption of fruits and vegetables was considered adequate when it was ≥ five times/day (29).
- Nutritional status: defined by the body mass index (BMI), which is given by the measurement of weight in kilograms divided by the square of the height in meters, and was classified according to criteria from the World Health Organization (WHO): ≤24.9 kg/m² = Eutrophy; ≥25.0 −29.9 kg/m² = Overweight; ≥30.0 kg/m² = Obesity (30). For the assessment of nutritional status, the body weight of each participant was measured using a portable analog scale with a precision of 100 g. Height was measured using a portable stadiometer with a precision of 1 mm. Both measurements were performed in duplicate and the respective mean values were considered.

### Statistical analyses

From the sample of 1,128 women, power calculations of the present study were performed using Stata 12.0 (StataCorp LP, College Station, TX, USA) software using the “sampsi” command. There was a power of 80% for associations with a prevalence ratio (PR) of 1.7 or greater and a confidence level of 95%.

Descriptive analyses were performed, using absolute and relative frequencies and the respective confidence intervals (95% CI), to establish multimorbidity patterns, investigating the most prevalent chronic condition dyads and triads, in addition to the prevalence of patterns among the population with multimorbidity, and their frequency in relation to age and nutritional status of women. Multivariable analysis was performed in two stages. Initially, considering that it was a complex sample, Poisson regression with robust variance was used to understand the relationship between the exposure variables and the identified multimorbidity patterns, and crude and adjusted analyses were performed for all dyads with a prevalence ≥ 2% considering three levels of determination (31). The first level included sociodemographic variables, the intermediate level included lifestyle variables, and the third-level variable was nutritional status. Subsequently, considering the possible confounding factors (p ≤ 0.20), obesity exposure was tested as an independent risk factor for the occurrence of multimorbidity patterns (with a prevalence ≥ 2%) through different adjustment models: Model I, with no adjustment; Model II with adjustment for sociodemographic variables; and Model III, with adjustment for sociodemographic and lifestyle variables. A significance level of 5% was considered for all the analyses.

## RESULTS

We interviewed 1,128 women, and 153 (11.94%) were classified as losses and refusals. The women were characterized in terms of their sociodemographic profile, lifestyle, and nutritional status (Table 1). Their mean age was 43.4 years (SD = 13.4) and mean schooling time was 9.8 years (SD = 10.8). There was a predominance of white women (74.5%), of those who lived with a partner (63.8%), who belonged to economic class C (53.1%), and who were employed (58.1%). Half of the population had a family income per capita of up to R$ 869.00, and 41.7% had 11 or more years of study. Regarding lifestyle characteristics, most reported inadequate food consumption of fruits and vegetables (56.3%), physical inactivity (85.6%), consumption of alcoholic beverages (66.8%), and not smoking (58.6%). Approximately one-third of the participants had obesity (32.9%).

**Table 1:** Sample characteristics and prevalence of 0-1 chronic condition, 2 chronic conditions and ≥3 chronic conditions according to sociodemographic and lifestyle characteristics. São Leopoldo, RS, Brazil, 2015 (n = 1,128)

Variables		n	Multimorbidity			p-value
			0-1 chronic condition n (%)	2 chronic conditions n (%)	≥3 chronic conditions n (%)	
Overall		1128	883 (78.3)	107 (9.5)	138 (12.2)	
Age (years)						<0.001^a^
	20-29	216	213 (98,6)	1 (0.5)	2 (0.9)	
	30-39	244	233 (95.5)	10 (4.1)	1 (0.4)	
	40-49	276	231 (83.7)	27 (9.8)	18 (6.5)	
	50-59	228	139 (61.0)	36 (15.8)	53 (23.3)	
	60-69	164	67 (40.9)	33 (20.1)	64 (39.0)	
Skin color						0.412^b^
	White	840	660 (78.6)	74 (8.8)	106 (12.6)	
	Brown	181	135 (74.6)	22 (12.2)	24 (13.3)	
	Black	84	67 (79.8)	11 (13.1)	6 (7.1)	
	Indigenous	11	10 (90.9)	0 (0)	1 (9.1)	
	Yellow	12	11 (91.7)	0 (0)	1 (8.3)	
Marital status						0.183^b^
	Not having a partner	408	319 (78.2)	32 (7.8)	57 (14.0)	
	Having a partner	720	564 (78.3)	75 (10.4)	81 (11.3)	
Education (years)						<0.001^a^
	≥11	470	409 (87.0)	30 (6.4)	31 (6.6)	
	8-10	199	162 (81.4)	19 (9.6)	18 (9.1)	
	5-7	253	186 (73.5)	33 (13.0)	34 (13.4)	
	0-4	204	126 (61.8)	24 (11.8)	55 (26.5)	
Economic class						0.580^a^
	A + B (high)	390	316 (81.0)	34 (8.7)	40 (10.3)	
	C	596	458 (76.9)	58 (9.7)	80 (13.4)	
	D + E (low)	136	106 (77.9)	14 (10.3)	16 (11.8)	
Household income *per capita* (quartiles)						0.165^a^
	I (low)	273	226 (82.8)	21 (7.7)	26 (9.5)	
	II	273	210 (76.9)	24 (8.8)	39 (14.3)	
	III	273	213 (78.0)	24 (8.8)	36 (13.2)	
	IV (high)	272	205 (75.4)	36 (13.2)	31 (11.4)	
Occupation						<0.001^b^
	Employed	654	578 (88.4)	39 (6.0)	37 (5.7)	
	Unemployed	472	303 (64.2)	68 (14.4)	101 (21.4)	
Consumption of fruits and vegetables						<0.001^b^
	Adequate	492	352 (71.5)	58 (11.8)	82 (16.7)	
	Inadequate	634	529 (83.4)	49 (7.7)	56 (8.8)	
Physical activity						0.203^b^
	Active	162	132 (81.5)	17 (10.5)	13 (8.0)	
	Inactive	966	751 (77.7)	90 (9.3)	125 (12.9)	
Alcohol consumption						<0.001^b^
	No	346	231 (66.8)	48 (13.9)	67 (19.4)	
	Yes	697	601 (86.2)	50 (7.1)	46 (6.6)	
Smoking status						<0.001^b^
	Non-smoker	661	532 (80.5)	62 (9.4)	67 (10.1)	
	Former smoker	259	177 (68.3)	32 (12.4)	50 (19.3)	
	Current smoker	208	174 (83.7)	13 (6.3)	21 (10.1)	
Nutritional Status						<0.001^a^
	Eutrophy	380	340 (89.5)	22 (5.8)	18 (4.7)	
	Overweight	373	285 (76.4)	40 (10.7)	48 (12.9)	
	Obesity	369	253 (68.6)	45 (12.2)	71 (19.2)	

a P-value of the chi-square test for linear trend.

b P-value of the chi-square test for heterogeneity of proportions.

The average of chronic conditions in the population studied was 0.9 (SD = 1.3), and among those who had some chronic morbidity, it was 1.9 (SD = 1.2). The individuals who presented zero to one, two, and three or more morbidities represented 78.3%, 9.5%, and 12.2%, respectively of the studied population. As can be seen in Table 2, eleven multimorbidity patterns were present in more than 2% of the study population, with the dyad “hypertension + dyslipidemia” being present in 6.4% of the general population studied and in almost 30% of the population with multimorbidity. Among the individuals with multimorbidity, chronic conditions with higher proportions were hypertension (71.4%), dyslipidemia (37.6%), and CMD (35.9%). It was observed that among the most prevalent chronic condition dyads, most were combined with hypertension. Other conditions that combined more frequently were dyslipidemia, CMD, acid-related digestive disorders (medicines for the alimentary tract, such as antacids and antispasmodics), DM, and circulatory disorders, which also comprised the most prevalent chronic condition triad, with “hypertension + dyslipidemia + circulatory disorders” present in about 2% of the study population. In addition, it was observed that among 11 dyads of the most prevalent conditions, five were constituted by conditions of the group that could be characterized by cardiometabolic conditions, including conditions such as hypertension, dyslipidemia, DM, and circulatory disorders.

**Table 2:** Chronic conditions, dyads and triads of chronic conditions most prevalent in the sample of women. São Leopoldo, RS, Brazil, 2015

Chronic conditions (prevalence ≥4%)		n	Prevalence in the sample (n = 1,128) % (95% CI)	Prevalence among the population with multimorbidity^a^ (n = 245) % (95% CI)
Hypertension		262	23.2 (20.8 – 25.8)	71.4 (65.3 – 77.0)
CMD		152	13.5 (11.5 – 15.6)	35.9 (29.9 – 42.3)
Acid-related digestive disorders		98	8.7 (7.1 – 10.5)	34.3 (28.4 – 40.6)
Dyslipidemia		97	8.6 (7.0 – 10.4)	37.6 (31.5 – 43.9)
Thyroid diseases		65	5.8 (4.5 – 7.3)	17.1 (12.6 – 22.5)
DM		60	5.3 (4.1 – 6.8)	22.9 (17.8 – 28.6)
Circulatory disorders		55	4.9 (3.7 – 6.3)	19.6 (14.8 – 25.1)
Chronic pain		45	4.0 (2.9 – 5.3)	13.1 (9.1 – 17.9)
Dyads				
	Hypertension + Dyslipidemia	72	6.4 (5.0 – 7.8)	29.4 (23.8 – 35.5)
	Hypertension + CMD	53	4.7 (3.5 – 6.1)	21.6 (16.6 – 27.3)
	Hypertension + Acid-related digestive disorders	53	4.7 (3.5 – 6.1)	21.6 (16.6 – 27.3)
	Hypertension + DM	45	4.0 (2.9 – 5.3)	18.4 (13.7 – 23.8)
	Hypertension + Circulatory disorders	41	3.6 (2.6 – 4.9)	16.7 (12.3 – 22.0)
	Acid-related digestive disorders + CMD	29	2.6 (1.7 – 3.7)	11.8 (8.1 – 16.6)
	Acid-related digestive disorders + Dyslipidemia	29	2.6 (1.7 – 3.7)	11.8 (8.1 – 16.6)
	Dyslipidemia + DM	27	2.4 (1.6 – 3.5)	11.0 (7.4 – 15.6)
	Dyslipidemia + Circulatory disorders	27	2.4 (1.6 – 3.5)	11.0 (7.4 – 15.6)
	Hypertension + Chronic pain	22	2.0 (1.2 – 3.5)	9.0 (5.7 – 13.3)
	CMD + Dyslipidemia	22	2.0 (1.2 – 3.5)	9.0 (5.7 – 13.3)
Triads			
	Hypertension + Dyslipidemia + Circulatory disorders	24	2.1 (1.4 – 3.2)	9.8 (6.4 – 14.2)
	Hypertension + Dyslipidemia + Acid-related digestive disorders	23	2.0 (1.3 – 3.0)	9.4 (6.0 – 13.8)
	Hypertension + Dyslipidemia + DM	21	1.9 (1.2 – 2.8)	8.6 (5.4 – 12.8)

95% CI: 95% Confidence Interval; CMD: Common mental disorders; DM: Diabetes Mellitus.

a Multimorbidity defined as two or more chronic conditions in the same individual.

**Figure 1:**
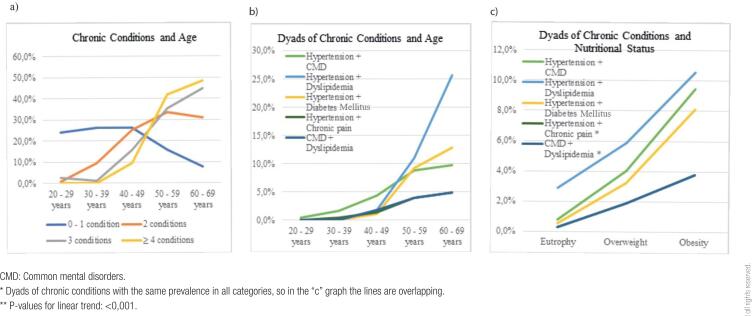
**a**) Number of chronic conditions in the sample of women according to age groups. **b**) Prevalence of dyads of chronic conditions in the sample of women according to age groups. **c**) Prevalence of dyads of chronic conditions in the sample of women according to nutritional status categories. São Leopoldo, RS, Brazil, 2015. (n = 1,128).

In Figure 1, graph “a” shows the number of chronic conditions according to the age groups of the study population, while graphs “b” and “c” show the prevalence of dyads of chronic conditions according to age and BMI, respectively. Graph “a” shows an abrupt and marked increase of four or more chronic conditions between 40 and 50 years of age, and multimorbidity reaching 10% of women between 30 and 39 years old. Graph “b,” on the other hand, shows an increase in the prevalence of all dyads of morbidities as age increases, although dyads containing CMD show some stabilization after 50 years of age. The dyad “hypertension + dyslipidemia” presents the most accentuated increase after 40 years of age. Regarding nutritional status, all multimorbidity patterns increase as the BMI categories increase, with greater magnitude for the dyad “hypertension + dyslipidemia” (graph c).

Table 3 shows that obesity is strongly associated with multimorbidity patterns involving the presence of conditions in the group of cardiovascular and metabolic diseases, such as hypertension, dyslipidemia, DM, and diseases related to mental health, such as CMD, as well as chronic pain. After adjustment in multivariable analysis, it was observed that obesity increased the probability of occurrence of “hypertension + CMD” and “dyslipidemia + CMD” in women in the sample by more than three times.

**Table 3:** Association between obesity and dyads of chronic conditions according to the different adjustment models. São Leopoldo, RS, Brazil, 2015. (n = 1,128)

		Model I		Model II		Model III	
		PR (95% CI)	p-value	PR (95% CI)	p-value	PR (95% CI)	p-value
Obesity (No = ref)							
	Hypertension + Dyslipidemia	2.41 (1.54 – 3.77)	<0.001	1.72 (1.12 – 2.64)	0.014^a^	1.79 (1.10 – 2.92)	0.018^e^
	Hypertension + CMD	3.97 (2.28 – 6.91)	<0.001	3.00 (1.73 – 5.20)	<0.001^b^	3.63 (1.94 – 6.78)	<0.001^f^
	Hypertension + DM	4.37 (2.35 – 8.15)	<0.001	3.00 (1.63 – 5.53)	<0.001^c^	2.87 (1.39 – 5.94)	0.004^f^
	Hypertension + Chronic pain	3.57 (1.51 – 8.44)	0.004	2.59 (1.11 – 6.03)	0.028^d^	2.54 (1.10 – 5.90)	0.029^g^
	CMD + Dyslipidemia	3.57 (1.51 – 8.44)	0.004	2.82 (1.16 – 6.88)	0.023^a^	3.69 (1.08 – 12.65)	0.038^h^

PR: prevalence ratio; 95% CI: 95% Confidence Interval; CMD: Common mental disorders; DM: Diabetes Mellitus

Model I: effect of obesity with no adjustment.

The following confounding factors were considered: Model II: effect of obesity adjusted for sociodemographic variables.

a adjusted for age and occupation;

b adjusted for age, education and income;

c adjusted for age, education and income;

d adjusted for age and education.

Model III: effect of obesity adjusted for sociodemographic and lifestyle variables.

e adjusted for alcohol consumption;

f adjusted for consumption of fruits and vegetables, physical activity and alcohol consumption;

g adjusted for physical activity;

h adjusted for consumption of fruits and vegetables and alcohol consumption.

## DISCUSSION

This study investigated the multimorbidity patterns in a representative sample of women aged 20-69 years and identified, among the most prevalent, eleven dyads and three triads of chronic morbidities, with hypertension and dyslipidemia being the conditions most present in the different patterns identified. The increase in age was related to the increase in the prevalence of all observed patterns, and although our hypothesis regarding lifestyle variables has not been confirmed, obesity was identified as the main modifiable risk factor for multimorbidity patterns that contained conditions related to the cardiovascular and metabolic system as well as mental health, in which their presence more than doubled the likelihood of these patterns occurring.

Our findings are in line with the findings of the National Health Survey (PNS), 2013 (32), constituted by a representative sample of the Brazilian adult population in which, among the 14 conditions evaluated, “hypertension + dyslipidemia” was the most prevalent dyad, while the most prevalent triad was “hypertension + dyslipidemia + DM,” which was also among the three most frequent conditions found in our study. However, the PNS showed that, among individuals with multimorbidity, 13.6% had “hypertension + dyslipidemia,” whereas in our population of women with multimorbidity, the prevalence of this combination was higher (29.4%). Methodological differences make comparability between studies difficult, and may explain, at least in part, the lower prevalence found in the PNS, since this cross-sectional survey included men and women aged ≥ 18 years from all regions of the country. It should be noted that the highest prevalence of multimorbidity was found in women and in the southern states of the country (32).

Our study showed that the occurrence of multimorbidity patterns became more expressive around the age of 45, consistent with a study carried out in Spain that included the presence of 146 chronic conditions in patients from primary health care. It was also observed that hypertension and dyslipidemia were among the most prevalent conditions in individuals with multimorbidity and that, from the age of 45 years, this was the most common combination (14).

This research revealed that almost half of the dyads of morbidities were constituted by conditions belonging to the cardiovascular and endocrine systems, in agreement with the literature, which showed cardiometabolic pattern among the most prevalent, and included combinations of conditions such as hypertension, dyslipidemia, DM, heart diseases, and obesity (4,7,33,34). In PNS 2013, the cardiometabolic pattern, composed of high blood pressure, acute myocardial infarction, angina, heart failure, stroke, dyslipidemia, DM, and arthritis/rheumatism was among the patterns identified that explained 92% of the total variance in principal component analysis (33). This is explained by the fact that cardiovascular and metabolic diseases, such as DM, share both pathophysiological pathways and risk factors (35,36), which is why it is plausible that they coexist, in addition to the fact that the accumulation of their risk factors over time contributes to the increase in prevalence with age.

This study also showed that CMD was present in 36% of women with multimorbidity, which is consistent with the literature showing that mental health conditions often coexist with other diseases (7,37–39). Furthermore, it was observed that among the most prevalent dyads of conditions, CMD appeared in combination with conditions such as hypertension and dyslipidemia, similar to that observed in the study by Bobo and cols. (2016). Nunes and cols. (2016) also found the dyad “hypertension + depression” among the most prevalent in the adult population, in a study conducted in southern Brazil (7). It has been shown in literature that a bidirectionality and dose-response association exists in the relationship between psychiatric disorders, such as depression, and conditions that are part of the metabolic syndrome, such as dyslipidemia and hypertension, in which interactions occur between multiple factors, including iatrogenic effects of psychotropic drugs, propensity to unhealthy lifestyles, and genetic and pathophysiological vulnerability, which explains our findings, in which these conditions combine with each other (40).

Our findings pointed out obesity as the main independent risk factor for most of the identified multimorbidity patterns, which included combinations of cardiovascular diseases, and among those with DMor CMD. In Australia, a cohort of women aged 45-50 years also found that obesity increased the chances of having a cardiometabolic pattern by almost 2.5 times, which was characterized by cardiovascular disease, DM, and glucose intolerance (18). PNS 2013 data, including men and women, showed four multimorbidity patterns with different characteristics from our findings, but they were also associated with obesity: cardiometabolic/cancer, mental/occupational, musculoskeletal, and respiratory patterns (41).

In relation to cardiometabolic diseases, the role of obesity can be explained because its occurrence requires functional adaptations of the systems, due to its implication in inflammation and vascular homeostasis, in addition to mediation with other cardiometabolic conditions among themselves (42), thus complicating the management and prognosis of these diseases (43). Regarding mental health diseases, evidence has shown an association between obesity and psychiatric disorders, such as anxiety and, mainly, depression (44,45), treating it as a bidirectional relationship, intertwined by a series of biological, psychological, and even behavioral mechanisms, which result in the fact that the presence of one condition increases the chance of the other occurring (45). This in turn would explain our results, since obesity appears as a risk factor for cardiovascular conditions in combination with CMD.

For some authors, obesity is considered a chronic condition in the investigation of multimorbidity (4,39). According to some others, it is considered a risk factor for determining chronic conditions (18,41). Our study treated obesity as a proximal determinant of different multimorbidity patterns and, in line with previous studies; it revealed a higher probability of multimorbidity among women with obesity compared to those without obesity, regardless of sociodemographic and behavioral variables.

Some aspects must be considered when interpreting our findings. Initially, the identification of chronic morbidities through ATC determined that these conditions were already established, since they had an indication for pharmacological treatment. However, the initial treatment indicated for many chronic conditions is non-pharmacological, such as the adoption of healthy habits. In this study, as women who were already using medicines were included, it is plausible to think that the isolated non-pharmacological treatment was not enough, suggesting the severity of the disease, and therefore, the indispensability of the medicines. Moreover, information on medicines use was reported, so our measure of chronic conditions may be underestimated, indicating that the prevalence of multimorbidity could be even higher. That's because it was not possible to identify chronic conditions in cases in which the woman was not using medicines at the time of the interview due to lack of access to treatment. But, in the literature it is already well established that the lack of access to pharmacological treatment is less than 5% (46). Yet, considering that we investigated the current use of medicines, this strategy did not allow captured diagnosed chronic conditions in non-pharmacological treatment. Our study is a cross-sectional design, and, therefore, the temporality bias must be considered. To minimize this bias, we used different adjustment models, with three levels of determination, to evaluate the association of potential risk factors on the occurrence of multimorbidity patterns. In addition, all adjustments in the multivariable models considered the collinearity between the independent variables, as well as the potential confounding factors associated with exposure and outcome (p ≤ 0.20) for each multimorbidity pattern analyzed, as a way of better understanding multicausality. Moreover, this study is population-based with a representative sample, which increases the possibility to generalize the results to the population of women in the investigated age group.

Finally, the study investigated young women and revealed the presence of common and important diseases in the identified dyads and triads patterns, whose low prevalence indicated an opportunity for prevention. These patterns were associated with a common risk factor, obesity, a global epidemic whose management is already a major public health challenge (47). The obesity epidemic has worsened with the pandemic of the coronavirus (COVID-19), in which social distance and self-quarantine that were defended in order to prevent further spread of the virus resulted in increase in body weight among other side effects (48). The scenario is even worse when evidence has already been shown about the negative impact of the covid-19 pandemic in relation to the occurrence of depressive symptoms and/or anxiety (49), at the same time that the prevalence of hypertension and other cardiovascular diseases has increased in the population (36,50). Furthermore, the increase in the prevalence of patterns is strongly related to aging, and although the adult population is still the largest population group (51), it is possible to speculate that, in the current scenario, a good portion of this population will have multimorbidity even before reaching old age.
